# F217. BASIC SELF-DISTURBANCE IN ADOLESCENCE AND SCHIZOPHRENIA-SPECTRUM DISORDERS IN YOUNG ADULTHOOD: A 7-YEAR FOLLOW-UP STUDY AMONG TREATMENT-SEEKING ADOLESCENTS

**DOI:** 10.1093/schbul/sby017.748

**Published:** 2018-04-01

**Authors:** Dan Koren, Yair Tzivoni, Liat Schalit, Noa Reznik, Merav Adres, Josef Parnas

**Affiliations:** 1 University of Haifa; 2 University of Copenhagen

## Abstract

**Background:**

Phenomenological research indicates that disturbance of the basic sense of self may be a core phenotypic marker of schizophrenia spectrum disorders. Basic self-disturbance refers to a disruption of the sense of first-person perspective and self-presence that is associated with a variety of anomalous subjective experiences. Recent cross-sectional and prospective pilot studies provided preliminary support for the notion that SD may provide a means of further “closing in” on individuals truly at high-risk for psychosis, particularly of schizophrenia spectrum disorders (SSD). The goal of this study was to replicate and extend these pilot findings by examining the long-term persistence of SD and the degree to which their level in adolescence predicts SSD seven years later in young adulthood.

**Methods:**

The 7-year stability of SD and their association with later in life SSD were explored in a sample of 40 young adults. SD was assessed with the Examination of Anomalous Self-Experience (EASE), prodromal symptoms and syndromes were assessed with the Structured Interview for Prodromal Syndromes (SIPS), present and lifetime diagnoses of schizophrenia-spectrum and other co-morbid disorders were assessed with the Kiddie Schedule for Affective Disorders and Schizophrenia (K-SADS) in adolescence and the Operational Criteria (OPCRIT) checklist for psychotic and affective illness in young adulthood, level of distress with the Mood and Anxiety States Questionnaire (MASQ), and psychosocial functioning with the Strength and Difficulties Questionnaire (SDQ).

**Results:**

Forty young adults (Mean age=23.7, S.D.=1.3) out of the 82 who had participated seven years earlier in a study on the association between SD and attenuated psychosis symptoms (APS) were available and agreed to participate in the 1-year follow-up (Mean=1.4, S.D.=0.8). There were no significant differences between those who were available and those who lost for the follow-up assessment on any of the major socio-demographic or clinical variables at baseline. Eight (20%) of the 40 participants in the present study met diagnostic criteria for an SSD (2 Schizophrenia, three non-organic psychotic disorder, and three schizotypal personality disorder). The total EASE score was slightly higher in young adulthood compared to seven years earlier. However, this can reflect a difference in the administration method of the EASE between the two occasions. Consistent with our first hypothesis, the correlation between the total EASE score at baseline and 7-year follow-up was moderate and significant (r=0.59, p<.001). Similarly, consistent with our second hypothesis, SD at baseline was a significant predictor of an SSD diagnosis in young adulthood.

**Discussion:**

These results provide further support for the temporal stability of SD over time. Also, they provide further support for the notion that SD is a phenotypic indicator of risk for SSD.

